# *Corynebacterium coyleae* as potential urinary tract pathogen

**DOI:** 10.1007/s10096-019-03565-4

**Published:** 2019-04-27

**Authors:** Beata Sokol-Leszczynska, Piotr Leszczynski, Dominika Lachowicz, Olga Rostkowska, Mariusz Niemczyk, Tomasz Piecha, Alex van Belkum, Anna Sawicka-Grzelak, Grazyna Mlynarczyk

**Affiliations:** 1 Department of Medical Microbiology, Medical University of Warsaw, Warsaw, Poland; 2 Department of Medical Microbiology, University Medical Center, Hospital of the Infant Jesus, Warsaw, Poland; 3 Infection Control Team, University Medical Center, Hospital of the Infant Jesus, Warsaw, Poland; 4 Department of Transplantation Medicine, Nephrology and Internal Medicine, Medical University of Warsaw, Warsaw, Poland; 5 Department of Immunology, Transplant Medicine and Internal Diseases, Medical University of Warsaw, Warsaw, Poland; 6 Department of General Oncological and Functional Urology, Medical University of Warsaw, Warsaw, Poland; 7 BioMérieux, R&D Microbiologie, Route de Port Michaud, 38390 La Balme les Grottes, France

**Keywords:** *Corynebacterium coyleae*, Urinary tract infection, Microflora, Antimicrobial susceptibility

## Abstract

*Corynebacterium coyleae* is part of the commensal microflora of the skin, urethra, mucous membranes, and genital tract. Isolates from patients with urinary tract infection (UTI) were reported, but the pathogenic potential of this species has not been defined yet. The aim of the study is to determine whether *C. coyleae* could be the etiological agent of UTI and to analyze its antibiotic susceptibility. Urine samples were cultured quantitatively according to accepted laboratory procedures. The identification of bacterial isolates was carried out using the Vitek MS (bioMérieux) and antibiotic susceptibility was tested using disc diffusion according to EUCAST guidelines. Between 1 January 2017 and 30 October 2018, a total of 39 *C. coyleae* strains were isolated. This represented 0.32% of all urine samples cultured in the laboratory during the collection period. The strains were isolated from samples obtained from 35 women and 3 men (age median for all—64 years). One female patient presented with *C. coyleae* in her urine twice at an interval of 21 months. In six cases of UTI, *C. coyleae* was isolated in monoculture. The isolates had the same resistance pattern. A total of 11 strains were obtained from cases with a clinical diagnosis of UTI. In 13 cases, the strain was cultured in a monoculture and in 28 cases with accompanying species. All strains were susceptible to vancomycin. However, resistance to ciprofloxacin was observed for 58.4% of the strains. Urine isolates of *C. coyleae* must be considered as contamination or normal flora in most cases (28/39, 72%). In the remaining cases, it can be considered as potential etiologic agents, mostly in women and especially in the 6 UTI cases where *C. coyleae* was found as the single culture-positive species. Several of these isolates demonstrate resistance to antibiotics commonly used in empiric treatment of urinary tract infections.

## Introduction

*Corynebacterium coyleae* was isolated for the first time in 1997 by Funke et al. [1] from the blood cultures of six patients with episodes of fever of unknown origin; one patient was infected with the human immunodeficiency virus (HIV), whereas the other five patients had a previous surgical intervention as the underlying condition. Furthermore, C. *coyleae* has been recovered from a definite sepsis case, probable case of sepsis and soft tissue infection, and possible post-transfusion bacteremia, neonatal bacteremia [2], pancreatic abscesses mimicking malignant neoplasm [3], burn injuries [4], pleural fluid specimens [1, 5], abscesses [6], and ulcers [7]. Still, there is ongoing controversy on its official pathogen status.

Resistance to beta-lactam antibiotics has not commonly been described in *C. coyleae* isolates. All isolates tested by Fernandez et al. [2] were susceptible to β-lactams, gentamicin, rifampin, tetracycline, vancomycin, linezolid, and resistant to clindamycin. Isolates from hospitalized patients reported by Barberis et al. [5] demonstrated a significantly higher resistance rate to antibiotics than those from outpatients. The aim of the current study is to determine whether *C. coyleae* could be an etiological agent of urinary tract infections and to analyze its antibiotic susceptibility.

## Material and methods

The retrospective analysis included urine cultures performed in 2017 (January 1st–December 31st) and in 2018 (January 1st–October 31st). The samples were taken from in- and outpatients. The patients were aged 21–90 (arithmetic mean—62 years, midrange—55 years, median—64 years). Urine samples were cultured quantitatively according to the laboratory’s procedures. Middle stream urine (MSU) samples from women after transplantation and catheterized samples (CS) were cultured with 10 μl loop on CPS medium (bioMerieux, France); samples from men were cultured with 1 μl loop; and 100 μl samples from percutaneous nephrostomy and samples taken directly from renal pelvis were cultured. CPS and McConkey were applied for MSU, and CS were additionally cultured on blood agar and Schoedler’s medium (bioMerieux, France). The samples were incubated in 37 °C for 18–24 h. The identification was initially based on the colony and cell morphology, and then carried out in the Vitek MS (bioMérieux, France). VITEK MS is an adequate tool for the identification of *C. coyleae*. Although the database only contains 37 spectra obtained for 9 strains, it is 97.3% accurate and does not provide incorrect identifications (no discordances). Due to the small size of *C. coyleae* colonies, circa 5–10 colonies were applied on the MALDI sample slide. The susceptibility was tested with the disc diffusion method according to EUCAST guidelines and standardized laboratory procedures.

## Results

A total number of 39 strains were isolated, representing 0.32% of all urine cultures during this period. Thirty-six strains were from women and three from men. In case of one female (73 years), *C. coyleae* was isolated twice at an interval of 21 months. In 2017, seven strains were cultured from patients with UTI symptoms; in three cases as a monoculture (10^4^ cfu/ml); in 3 cases together with *Enterococcus faecalis* (10^3^ cfu/ml twice, 10^5^ cfu/ml once); and in one case with *E. coli* (10^4^ cfu/ml); 17 strains were cultured from non-UTI patients; in 9 cases as monoculture (twice 10^5^ cfu/ml, fivefold 10^4^ cfu/ml, once 10^3^ cfu/ml and once 10^2^ cfu/ml). In 2018, 4 strains were cultured from patients with UTI symptoms cases; in 3 cases as monoculture (once 10^4^ cfu/ml, twice 10^3^ cfu/ml) and in one case with *E. coli* (10^2^ cfu/ml); 11 strains from non-UTI patients; 5 strains presented as monocultures (thrice 10^4^ cfu/ml and twice 10^3^ cfu/ml) and in 6 cases another species was found in addition (twice *E. coli* 10^3^ cfu/ml, twice *E. faecalis* 10^4^ cfu/ml, once *S. agalactiae* 10^4^ cfu/ml and once *S. hemolyticus* 10^3^ cfu/ml) (Tables 1 and 2).

**Table 1:** Bacterial isolates accompanying *C. coyleae* from 11 patients with suspected UTI

Patient	Isolates	
	*C. coyleae*	Accompanying species
	Bacteriuria (cfu/ml)	
1	10^3^	None
2	10^3^	
3	10^4^	
4	10^4^	
5	10^4^	
6	10^4^	
7	10^4^	*E. faecalis* (10^3^)
8	10^4^	*E. faecalis* (10^3^)
9	10^4^	*E. faecalis* (10^5^)
10	10^3^	*E. coli* (10^2^)
11	10^4^	*E. coli* (10^4^)

**Table 2:** *C. coyleae* isolates from non-UTI (control) 28 cases

Patient	Isolates	
	*C. coyleae*	Accompanying species
	Bacteriuria (cfu/ml)	
1	10^2 *^	None
2	10^3^	
3	10^3^	
4	10^3^	
5	10^4^	
6	10^4^	
7	10^4^	
8	10^4^	
9	10^4^	
10	10^5^	
11	10^5^	
12	10^4^	
13	10^4^	
14	10^4^	
15	10^3^	*P. mirabilis* (10^3^)
16	10^4^	*E. faecalis* (10^3^)
17	10^4^	*E. faecalis* (10^3^)
18	10^4^	*S. epidermidis* (10^4^)
19	10^4^	*S. saprophyticus* (10^4^)
20	10^4^	*E. coli* (10^5^)
21	10^4^	*P. vulgaris (*10^5^)
22	10^5^	*S. hominis (*10^4^)
23	10^3^ (predominant)	*E. coli* (10^3^) + *C. albicans* (10^4^)
24	10^5^	*E. coli* (10^3^)
25	10^3^	*S. haemolyticus* (10^3^)
26	10^3^	*E. faecalis* (10^4^)
27	10^4^	*E. faecalis* (10^4^)
28	10^5^	*S. agalactiae* (10^4^)

*– urine obtained by catheterization

In one case, two urine samples were taken on the same day in different ways: middle stream urine (MSU) and material directly from the nephrostomy. Both *C. coyleae* and *E. coli* were isolated from MSU and only *E. coli* from the renal pelvis. In the other case, the following organisms were cultured: *C. coyleae*, *Proteus mirabilis*, *Prevotella* spp., *Peptococcus asaccharolyticus*, *Lactobacillu*s spp., and *S. epidermidis* from the material obtained by nephrostomy from a patient with pyonephrosis. Three days after antibiotic therapy was started, only *P. mirabilis* was cultured from the urine obtained from nephrostomy (Table 3).

**Table 3:** Bacterial strains isolated from urine samples taken from two patients

Patient	Urine sample	Isolates/bacteriuria (cfu/ml)
Female, 73 years, UTI	Middle stream urine	*C. coyleae* (10^4^) *E. coli* (10^5^)
	Nephrostomy	*E. coli* (10^2^)
Female, 26 years, UTI susp.	Nephrostomy (pyonephrosis)	*C. coyleae* (10^3^) *P. mirabilis* (10^3^) *S. epidermidis* (10^3^) *Prevotella* spp. (10^5^) *P. assacharolyticus* (10^5^) *Lactobacillus* spp. *(*10^5^)
	Nephrostomy (sample obtained 3 days later)	*P. mirabilis* (10^5^)

We analyzed the susceptibility of all strains to antibiotics applied in the treatment of UTI. Susceptibility rate to benzylpenicillin, ciprofloxacin, gentamycin, tetracycline, and vancomycin was 5.1%, 43.6%, 69.2%, 87.2%, 100%, respectively (Table 4).

**Table 4:** Antimicrobial susceptibility of *C. coyleae* isolates

Antibiotic susceptibility of *C. coyleae* isolates					
	Benzylpenicillin	Ciprofloxacin	Gentamycin	Tetracycline	Vancomycin
Our study (*n* = 39)	5.1%	43.6%	69.2%	87.2%	100%
Fernandez et al. [2] (*n* = 12)	8.3%	83.3%	100%	100%	100%

## Discussion

We present the biggest collection of clinical *C. coyleae* strains in current literature. The pathogenic role of this individual *Corynebacterium* species in human infections and their mechanisms of pathogenesis remain to be elucidated. There is a clear lack of distinction between colonization and infectious status, and there remains a need for additional clinical studies as well [6, 7]. The results of studies performed by other researchers show that *Corynebacterium* species, particularly those taxa found as part of the normal microflora, are prominent contaminants of clinical materials, although occasionally it is difficult to correctly decide if presence of such bacteria implies contamination or has clinical relevance. If *Corynebacterium* spp. are recovered from urine specimen as the only bacterial species encountered with a titer of > 10^4^ cfu/ml or if it is the predominant organism recovered (> 10^5^ cfu/ml), it is recommended to identify the isolate to the species level [6]. In Table 5, we present recovery of *C. coyleae* isolates from the genitourinary tract of women and men and their clinical significance as demonstrated by various authors. In addition, Fernández-Natal et al. [2] reported *C. coyleae* isolates from cases of sepsis, soft tissue infection, and post-transfusion bacteremia, which in vitro were susceptible to beta-lactams, gentamicin, rifampin, tetracycline, vancomycin, and linezolid, and resistant to clindamycin (Table 4). Resistance to erythromycin occurred in 83.3% of isolates, all of them presented phenotypic cMLS and harbored the gene *erm*X. Ortiz-Perez et al. [8] also detected *erm*X in 8/12 strains. The most concerning fact is that a large percentage of our strains is resistant to ciprofloxacin. We agree that the empirical use of quinolones should not be recommended, and clinically relevant *Corynebacterium* species always require antimicrobial susceptibility testing [5].

**Table 5:** Literature review on *C. coyleae* isolations from genitourinary tract and their clinical significance

Literature	Clinical significance
Funke et al. (1997) [1]	Females with overactive bladder syndrome
Verhelst et al. (2006) [9]	*C. coyleae* occurred in 0.2% of vaginal microflora samples of healthy women (with grade 1 of Gram stain vaginal smears)
Turk et al. (2007) [10]	*C. coyleae* occurred in the semen of men with prostatitis with severe (> 1 million WBC/ml semen) and moderate (0.2–1 milion WBC/ml semen) inflammation as well as in semen from controls
Pearce et al. (2014) [11]	*C. coyleae* was most frequently cultured from urine samples from women with urgency urinary incontinence (UUI) than healthy controls
Yang (2015) [12]	Occurrence in vaginal microflora across gestation in pregnant women treated with placebo or probiotics
Brubaker et al. (2016) [13]	The statistical association between UUI symptoms and *C. coyleae* was demonstrated
Schwaderer et al. (2017) [14]	Urine from women with urinary tract stones
Barberis et al. (2018) [5]	Total nephrectomy following *C. coyleae* UTI in 36-year-old woman with a history of chronic kidney failure under hemodialysis due to polycystic kidney disease, with several episodes of UTI

Contamination of a urine sample can contribute to under- or over-diagnosis of bacteriuria. In case that *C. coyleae* is a part of the normal microflora, it can potentially contribute to the implementation of unnecessary antimicrobial therapy [7]. In cases when the presence of *C. coyleae* in urine from a patient with UTI symptoms is accompanied by more commonly occurring pathogens (for example *E. coli*, *Enterococcus* spp., *S. saprophyticus* or *Proteus* spp.), it might be considered as contamination. However, if it comes to isolation of *C. coyleae* in quantities of 10^4^ cfu/ml or 10^5^ cfu/ml, the interpretation might be difficult, especially if the isolate is resistant to commonly prescribed antibiotics.

Based on the new findings presented here and the prior data published by others, *C. coyleae* is a rarely isolated species that should still be considered a pathogen that can be involved in complicated urinary tract infections, mostly in women. Isolation of *C. coyleae* may require confirmation by urinary tract punctuate to exclude contamination. Further studies on the role of *C. coyleae* in UTI are needed.
